# Compound heterozygous *RMND1* gene variants associated with chronic kidney disease, dilated cardiomyopathy and neurological involvement: a case report

**DOI:** 10.1186/s13104-016-2131-2

**Published:** 2016-06-27

**Authors:** Asheeta Gupta, Isabel Colmenero, Nicola K. Ragge, Emma L. Blakely, Langping He, Robert McFarland, Robert W. Taylor, Julie Vogt, David V. Milford

**Affiliations:** Birmingham Childrens Hospital, Steelhouse Lane, Birmingham, B4 6NH UK; Clinical Genetics Unit, West Midlands Regional Genetics Service, Birmingham Women’s Hospital, Birmingham, B15 2TG UK; Faculty of Health and Life Sciences, Oxford Brookes University, Oxford, OX3 0BP UK; Wellcome Trust Centre for Mitochondrial Research, Institute of Neuroscience, The Medical School, Newcastle University, Newcastle upon Tyne, NE2 4HH UK

**Keywords:** Mitochondrial disease, Chronic kidney disease, Global developmental delay, Sensorineural hearing loss, *RMND1* gene

## Abstract

**Background:**

Nuclear gene mutations are being increasingly recognised as causes of mitochondrial disease. The nuclear gene *RMND1* has recently been implicated in mitochondrial disease, but the spectrum of pathogenic variants and associated phenotype for this gene, has not been fully elucidated.

**Case presentation:**

An 11-month-old boy presented with renal impairment associated with a truncal ataxia, bilateral sensorineural hearing loss, hypotonia, delayed visual maturation and global developmental delay. Over a 9-year period, he progressed to chronic kidney disease stage V and developed a dilated cardiomyopathy. Abnormalities in renal and muscle biopsy as well as cytochrome *c* oxidase activity prompted genetic testing. After exclusion of mitochondrial DNA defects, nuclear genetic studies identified compound heterozygous *RMND1* (c.713A>G, p. Asn238Ser and c.565C>T, p.Gln189*) variants.

**Conclusion:**

We report *RMND1* gene variants associated with end stage renal failure, dilated cardiomyopathy, deafness and neurological involvement due to mitochondrial disease. This case expands current knowledge of mitochondrial disease secondary to mutation of the *RMND1* gene by further delineating renal manifestations including histopathology. To our knowledge dilated cardiomyopathy has not been reported with renal failure in mitochondrial disease due to mutations of *RMND1*. The presence of this complication was important in this case as it precluded renal transplantation.

## Background

Mitochondrial disorders are amongst the most common inborn errors of metabolism, with an estimated prevalence of 1 in 5000 live births [1]. Renal involvement is common either in isolation or as part of a multisystem disease. Knowledge of the genetic basis to mitochondrial disease has progressed significantly over the past 5 years and continues to evolve [2, 3]. It is now recognised that these diseases can be caused by nuclear as well as mitochondrial gene mutations, thus maternal inheritance patterns are not always seen. The ongoing identification of pathogenic nuclear mutations has broadened the spectrum of nuclear mitochondrial disease [2].

Mitochondrial cytopathy due to nuclear gene mutations is becoming increasingly more important for nephrologists to recognise since there may be potential for reversibility of some conditions that are caused by coenzyme Q10 biosynthesis defects [3] as well as the implication for treatment options, including renal transplant.

Several nuclear gene mutations have been identified in mitochondrial cytopathies with renal involvement [4, 5]. Renal involvement in these cases usually manifests as nephrotic syndrome or tubulopathies [4, 5]. Chronic kidney disease is a less common phenotype of nuclear mitochondrial gene mutations and has been reported in cases caused by mutations in *COQ2, SARS2* and recently in *RMND1* genes. Mutation of *COQ2* causes a Coenzyme Q10 (ubiquinone) deficiency and steroid resistant focal segmental glomerulosclerosis (FSGS) leading to end stage renal failure [6]. The *SARS2* gene is thought to be involved in mitochondrial translation. Gene mutations in *SARS2* result in hyperuracaemia, pulmonary hypertension, infantile renal failure and alkalosis (HUPRA syndrome) [7, 8]. Recently, the nuclear encoded *RMND1* gene has been implicated in causing mitochondrial disease of varying severity, manifesting in profound deafness and neurological involvement with or without renal and cardiac manifestations [9, 10]. The specific cardiac manifestations have not been published. Renal disease has comprised of renal dysplasia [2], renal tubular acidosis [2] and chronic kidney disease associated with proteinuria [9, 10]. Underlying histopathological diagnosis is yet to be described in these patients with chronic kidney disease. Though specific function of the RMND1 protein is not completely known; it is encoded by a nuclear gene and interestingly, has also been proposed to be essential for mitochondrial translation [11, 12].

We describe the natural history of a child’s phenotype associated with mutations in a nuclear gene implicated in mitochondrial protein synthesis causing mitochondrial disease with renal, cardiac and neuromuscular involvement.

## Case presentation

An 11-month-old Caucasian boy was referred to this department for investigation of a raised urea and creatinine. He was born at 37 weeks’ gestation to unrelated parents. He had difficulties with bottle-feeding in the first 2 weeks of life and at the time of weaning he was noted to choke on solids. He was also found to have bilateral profound sensorineural hearing loss on routine screening, which led to him receiving cochlear implants at 18 months. At 8 weeks, he was not fixing and following and was noted to be hypotonic. At 10 months, he became unwell with fever, lethargy and rash. His urea was 44 mmol/L but reduced to 14 mmol/L after rehydration therapy.

**Fig. 1:**
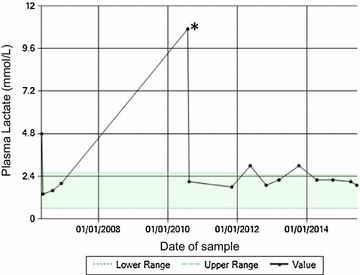
Plasma lactate profile from 11 months of age (time of presentation to nephrology) to present. The markedly raised plasma lactate level in 2010 (indicated by an *asterisk*) was during a period of transient inter-current viral illness

At the time of his referral he had global developmental delay, truncal hypotonia, delayed visual maturation and bilateral sensorineural hearing loss. He was not on any regular medication. Results of initial blood and urine tests together with serial plasma lactate levels from presentation are shown in Fig. 1 and Table 1 respectively.

**Table 1:** Initial blood and urine results taken at 11 months of age

Test	Result	
Blood		
Sodium	135 mmol/L	(135–145 mmol/L)
Potassium	5.4 mmol/L	(3.5–5.5 mmol/L)
Urea	*20.9* mmol/L	(*2.0*–*6.2* mmol/L)
Creatinine	*72* micromol/L	
Adjusted calcium	*2.58* *mmol/L*	(*2.1*–*2.5* *mmol/L*)
Phosphate	1.57 mmol/L	(1.3–2.3 mmol/L)
Bicarbonate	*18.8* mmol/L	(*23*–*29* *mmol/L*)
Magnesium	*1.45* *mmol/L*	(*1.6*–*2.6* mmol/L)
Total bilirubin	<5 micromol/L	(<5 micromol/L)
Alkaline phosphatase	*474* *IU/L*	(*110*–*320* *IU/L*)
Alanine transferase	21 IU/L	(6–45 IU/L)
Albumin	43 g/L	(35–50 g/L)
Ammonia	25 micromol/L	(22–48 micromol/L)
Lactate	*4.8* *mmol/L*	(*0.2*–*2.0* *mmol/L*)
Parathyroid hormone	*114* *nanogram/L*	(*10*–*65* *nanogram/L*)
Urine		
Urine protein/creatinine	16 mg/mmol	(<50 mg/mmol)
Urine albumin/creatinine	<1.93 mg/mmol	(<1.93 mg/mmol)
Urine retinol binding protein	<2.0 mg/L	(<2.0 mg/L)
Urine amino acids and organic acids	Normal	
Other		
Cerebrospinal fluid lactate	2.2 mmol/L	0.6–2.2 mmol/L

Abnormal results are highlighted in italics

**Fig. 2:**
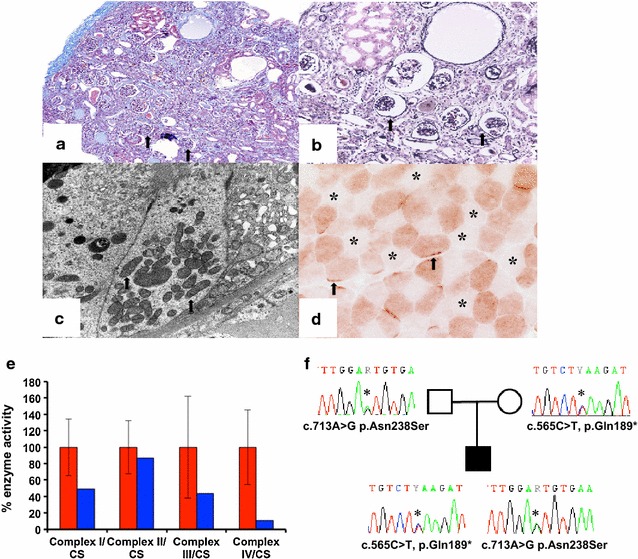
**a**,** b** Renal biopsy taken at time of presentation (11 months of age) showing moderate tubulointerstitial damage and numerous immature glomeruli (*arrow*) (**a**). Masson trichrome, ×100 (**b**) (*silver,* ×200). **c** Electron Micrograph of renal biopsy: A tubular epithelial cell showing mildly enlarged mitochondria with a fluffy granular matrix (*arrow*). **d** Assessment of oxidative enzyme histochemistry in a muscle biopsy revealed a mosaic pattern of cytochrome *c* oxidase (COX) activity. Presence of fibres with subsarcolemmal aggregates of mitochondria (*arrow*) and some type II fibres devoid of activity (*asterisk*) (×600). **e** The assessment of individual respiratory chain enzyme activities in muscle identified a combined OXPHOS deficiency in the patient (*blue bars*) compared to controls (*red bars*); mean enzyme activities shown for muscle controls (n = 25) are set at 100 %. The activities of complexes I and IV were both severely decreased with complex III activity decreased to a lesser extent, and evidence of complex II activity being spared. **f** Familial segregation of the identified compound heterozygous *RMND1* variants, a paternally-inherited p.Asn238Ser variant and a maternally-inherited p.Gln189* variant

A renal ultrasound scan showed right and left renal lengths were 6.2 cm (30th centile) and both kidneys were echogenic. The presence of renal impairment and echogenic kidneys on ultrasound prompted renal biopsy, which showed significant tubulointerstitial damage with immature glomeruli (Fig. 2a, b). Electron microscopy of renal tissue revealed abnormal mitochondria affecting the tubular cells (Fig. 2c). Assessment of oxidative enzyme histochemistry in a muscle biopsy revealed a mosaic pattern of cytochrome *c* oxidase (COX) activity (Fig. 2d), which was confirmed by direct measurement of respiratory chain enzyme activities; complexes I and IV were both severely decreased with complex III activity decreased to a lesser extent, and evidence of complex II activity being spared (Fig. 2e). Taken together, these data are consistent with a generalised disorder of mitochondrial function.

Brain magnetic resonance imaging (MRI) of the proband was reported to show abnormal signal in the cerebral white matter particularly in the temporal and subcortical white matter in the frontal lobes and abnormal signal in the periventricular white matter around the trigone and frontal horns. A cystic area was also seen in the temporal lobe. The features were in keeping with a metabolic condition or megalencephalic leukoencephalopathy with subcortical cysts.

Abnormal findings from kidney and muscle biopsy prompted genetic testing for mitochondrial disease. Diagnostic mitochondrial DNA (mtDNA) studies, including assessment of mtDNA copy number, mtDNA rearrangements and sequencing of the entire mitochondrial genome were all normal (data not shown). Due to a strong clinical suspicion of mitochondrial disease, nuclear genetic studies were undertaken, prioritising the analysis of the *RMND1* gene because of the clinical presentation. Direct Sanger sequencing of the *RMND1* coding exons in blood genomic DNA (primer sequences and conditions available on request from the authors) identified two heterozygous *RMND1* (GenBank Accession number NM_017909.2) variants predicted to be deleterious—a rare c.713A>G, p.Asn238Ser missense change (21/120626 alleles on ExAC and 5/12982 alleles on ESP6500) affecting a highly conserved residue and a novel c.565C>T, p.Gln189* truncating mutation. Analysis of parental blood DNA samples confirmed the mother carried the c.713A>G, p.Asn238Ser variation and the father carried the c.565C>T, p.Gln189* variation, thus demonstrating recessive inheritance (Fig. 2f). The p.Asn238Ser mutation has recently been shown by others to be a pathogenic RMND1 variant [10]. During this period of investigation the patient was commenced on Coenzyme Q10 supplementation. Despite starting this supplement our proband remained globally delayed in terms of his development.

**Fig. 3:**
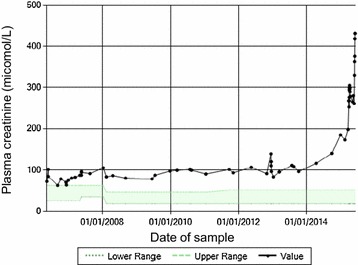
Plasma creatinine profile from the time of presentation

This child demonstrated stable chronic kidney disease (CKD) for 9 years since presentation. Plasma creatinine started to increase at the age of 10 years, indicating a decline in renal function (Fig. 3). At this time he was hospitalized for a gastroenteritic illness and cardiovascular examination revealed peripheral oedema and a gallop rhythm on auscultation. His echocardiogram confirmed a diagnosis of a dilated cardiomyopathy (ejection fraction of 35 %) for which he was given diuretics. His renal function failed to improve and he has commenced peritoneal dialysis.

## Conclusion

We report the case of a child found to harbour compound heterozygous variants in the *RMND1* gene, who presented with global developmental delay, truncal hypotonia, bilateral sensorineural hearing loss and renal impairment. He had stable CKD, but later had deterioration in renal function and required dialysis complicated by a dilated cardiomyopathy.

*RMND1* gene mutations have been linked to mitochondrial disease with varying severity and variable multisystem involvement [2, 9–13]. However, all cases presented with deafness and some element of neuromuscular disease. Of the published cases to date, seven had additional renal involvement and presented at or earlier than 18 months of age. This comprised of renal dysplasia in two patients homozygous for c.1349G>C, p.450Serext*32 stop-extension *RMND1* mutation, one of which also had cardiac involvement [2]. The same homozygous mutation was found in a third patient with renal tubular acidosis, cardiac involvement and seizures who died at the age of 10 years. Further details on the type of cardiac involvement have not been published [2]. A further case found to have renal tubular acidosis and seizures had a different compound heterozygous mutation (c.713A>G, p.Asn238Ser/c.829_830 + 2delGAGT, p.Glu277Glyfs*2). Three cases presented with renal failure and compound heterozygous mutations involving c.713A>G p.Asn238Ser together with other different mutations, as in our case. Two of these cases presented at birth, one with severe myopathy, dysautonomia, lactic acidosis, deafness and renal failure. This case also had abnormal brain MRI findings with delayed myelination and abnormal post periventricular white matter signal [9]. The other presented with hypotonia, intellectual disability deafness, renal failure and abnormal brain MRI with loss of white matter in the temporal lobes [10]. The final case presented at 14 months with hypotonia, intellectual disability, lactic acidosis, deafness and renal failure with a normal MRI brain [10]. The latter two patients were sisters. Both developed CKD and hypertension in the first decade of life. Both had proteinuria, but it is not known to what extent. Renal ultrasound revealed normal sized kidneys with normal echogenicity. Unfortunately neither underwent renal biopsy. They both underwent renal transplant successfully. Our proband did not have proteinuria and presented with renal impairment in infancy, with a very slow progression to ESRF. The development of dilated cardiomyopathy prevented renal transplant, which was being considered prior to this point. It is interesting that none of the three patients with renal failure have any reported cardiac involvement [9, 10].

Mitochondrial diseases classically vary in their severity, rate of progression and system involvement. Those with severe multisystem involvement and poor prognosis often present early in childhood (although there are exceptions) and most of these cases do not survive beyond childhood. Cases presenting later in childhood or adulthood often have less severe phenotypes and are more likely to be associated with mtDNA point mutations. Some individuals may have isolated renal involvement at presentation and have a better prognosis, for example those with the m.3243A>G point mutation, who often present later with proteinuria and go on to be diagnosed with FSGS [14]. These individuals may survive into adulthood, but many require renal replacement therapy and can be successfully transplanted [15, 16].

Sadly, a major characteristic of mitochondrial cytopathy is the unpredictability and progressive nature of systemic involvement. Rotig et al. [17] reported a child with mitochondrial cytopathy due to a nuclear gene mutation affecting the coenzyme Q_10_ pathway. This patient had bilateral sensorineural deafness, nystagmus and nephrotic syndrome that led to end stage renal failure (ESRF) but who successfully underwent a renal transplant. The patient subsequently suffered neurological deterioration after transplantation rendering him unable to walk; this was successfully treated with ubiquinone. Hameed et al. [18] described a 6 year old boy with hypoparathyroidism, muscle weakness, sensorineural deafness and FSGS who progressed to established renal failure and who was successfully dialysed and transplanted, but suffered a neurological deterioration 4 years later. This child was clinically diagnosed with mitochondrial cytopathy and he later died of respiratory failure from progressive muscle weakness. These cases highlight the complex issues of treating established renal failure in children with mitochondrial cytopathies because they may subsequently develop life-limiting complications as a result of disease progression. However, given the rate of progression in mitochondrial disease is variable and unpredictable, organ transplantation should be considered in the overall context of health needs and issues. Where possible, genetic confirmation and expert clinical advice on the mitochondrial disease course would be important contributions to the decision-making process.

Our patient presented at a young age and had a clinical diagnosis of a mitochondrial disease that has only recently been genetically defined. He has shown a gradual decline in renal function over the past 9 years. Unfortunately, he developed a cardiomyopathy with a poor ejection fraction of 35 %, which precludes renal transplant and has raised difficult clinical and ethical issues regarding his long-term renal replacement treatment. The genetic testing in this case was revisited after the initial panel of known mutations was negative. Identifying the genetic mutation not only helped to confirm the diagnosis but also in decision making regarding therapeutic options and counseling the family regarding prognosis. Hence, with expanding knowledge of new mutations linked to mitochondrial disease pursuing a genetic diagnosis in cases where there is a clinical suspicion is worthwhile.

We describe the phenotype of a child with novel compound heterozygous *RMND1* gene variants and mitochondrial disease causing multisystem failure including renal failure and cardiomyopathy. This report emphasizes the value of pursuing a genetic diagnosis in such cases to aid decisions regarding therapeutic options and counseling of patients and their families.

## Abbreviations

CKD: chronic kidney disease
COX: cytochrome *c* oxidase
DNA: deoxyriboneucleic acid
mtDNA: mitochondrial DNA
FSGS: focal segmental glomerulosclerosis
ESRF: end stage renal failure
